# Usability of deep learning and H&E images predict disease outcome-emerging tool to optimize clinical trials

**DOI:** 10.1038/s41698-022-00275-7

**Published:** 2022-06-15

**Authors:** Talha Qaiser, Ching-Yi Lee, Michel Vandenberghe, Joe Yeh, Marios A. Gavrielides, Jason Hipp, Marietta Scott, Joachim Reischl

**Affiliations:** 1 Precision Medicine and Biosamples, Oncology R&D, AstraZeneca, Cambridge, UK; 2 AetherAI, Taipei City, Taiwan; 3 Early Oncology, Oncology R&D, AstraZeneca, Cambridge, UK

**Keywords:** Computational biology and bioinformatics, Cancer imaging

## Abstract

Understanding factors that impact prognosis for cancer patients have high clinical relevance for treatment decisions and monitoring of the disease outcome. Advances in artificial intelligence (AI) and digital pathology offer an exciting opportunity to capitalize on the use of whole slide images (WSIs) of hematoxylin and eosin (H&E) stained tumor tissue for objective prognosis and prediction of response to targeted therapies. AI models often require hand-delineated annotations for effective training which may not be readily available for larger data sets. In this study, we investigated whether AI models can be trained without region-level annotations and solely on patient-level survival data. We present a weakly supervised survival convolutional neural network (WSS-CNN) approach equipped with a visual attention mechanism for predicting overall survival. The inclusion of visual attention provides insights into regions of the tumor microenvironment with the pathological interpretation which may improve our understanding of the disease pathomechanism. We performed this analysis on two independent, multi-center patient data sets of lung (which is publicly available data) and bladder urothelial carcinoma. We perform univariable and multivariable analysis and show that WSS-CNN features are prognostic of overall survival in both tumor indications. The presented results highlight the significance of computational pathology algorithms for predicting prognosis using H&E stained images alone and underpin the use of computational methods to improve the efficiency of clinical trial studies.

## Introduction

Survival (or time-to-event) analysis of cancer patients acts as a key driver for clinical decision-making and treatment planning^1,2^. Clinical trials in oncology stratify patients into different treatment subgroups based on biomarker/prognosis data and analyze survival in these subgroups^3,4^. The impact of patient(s) response to a certain treatment or therapy may also be observed by monitoring survival periods or time to disease progression. Objective estimation of survival probability may assist clinicians to personalize the treatment options and improve the selection of participants for conducting successful clinical trials^5^. Survival analysis is generally performed using genomic and protein (immunohistochemistry, IHC) biomarkers along with other clinical and patient characteristics or demographic information, such as age, gender, BMI, ethnicity, etc. Another relatively new but emerging direction is the tissue-based prognosis of the tumor microenvironment (TME), which consists of composite structures of normal, malignant cells, connective tissue infiltrated with immune cells and vessels. There is increasing evidence suggesting different components of TME structure influence tumorigenicity^6,7^ and previously shown to be prognostic in multiple cancers^6,8,9^.

In routine practice, cancer tissue slides are manually examined by a trained histopathologist under a microscope. The primary objective of the visual examination is to diagnose cancer by analyzing the morphological variations within the cancerous regions, quantifying the density of malignant areas, and observe the spatial arrangement of the TME^10^. However, careful visual examination of tissue slides is resource and time intensive, and the subjective nature of the histological practices inevitably leads to inter- and even intra-observer variability^11,12^. Recent advances in computational pathology enable the use of artificial intelligence and machine learning (AI&ML) algorithms to predict relevant clinical outcomes from histology image data. Automated algorithms are generally based on the concepts of digital image analysis which can analyze images to improve the precision and reproducibility in cancer diagnostics. The integration of artificial intelligence with whole slide imaging (WSIs) data sets of hematoxylin and eosin (H&E) stained tissue images have shown potential in linking complex associations of histology data with patient outcomes^13^. Related applications include the use of AI in immuno-oncology (IO) to quantify tumor mutual burden (TMB) and PD-L1 immunohistochemistry (IHC)^14,15^. Due to the rise of deep learning and the availability of scanned tissue slides, imaging-based prognosis is gaining more attention^16^.

Despite the overwhelming increase of deep learning related approaches for histopathology image analysis, there are several challenges that may hinder the development of these algorithms for routine clinical practice. Computational pathology algorithms usually require precisely annotated tissue regions to train AI&ML models and predict the slide label. In most of the real-world problems, and for the task at hand, the ground-truth (GT) label for overall survival is generally provided at the patient level and there are no detailed annotations provided about which regions of interest (ROIs) from the tissue slides are more likely to impact survival. Amongst existing approaches, the most common strategy is to select ROIs of a WSI (mainly from tumor component areas) identified by a pathologist or from a third-party application and train a supervised learning model to predict survival^17–23^. One important limitation is that such approaches introduce an inevitable bias to the model predictions and learn the prognostic features only from annotated ROIs. Therefore, the prognostic significance of the entire TME spatial organization remains largely unexplored. Another potential limitation is reusability, since, for larger data sets, it may not be feasible to attain detailed annotated regions of gigapixel images for training the model. This leads to our hypothesis that AI models can be trained without regions-level annotations and solely on patient level survival data to highlight the prognostic significance of different components of TME from H&E stained WSIs.

In this paper, we present a deep learning framework that leverages the patient level survival statistics to predict overall survival (OS) without the need for precisely annotated regions. Our framework demonstrates the usability of weakly supervised learning and visual attention mechanism to infer relevant spatial signatures for predicting cancer outcome without prior knowledge of tissue composite structures. This approach may also improve our understanding of disease pathogenetic mechanism and lead towards an unbiased objective prognosis. We demonstrate the significance of our prognosis framework on two cohorts of multi-center data sets including urothelial bladder carcinoma and lung cancer data from the publicly available National Lung Screening Trial (NLST) data set. The heterogeneous TME characteristics of urinary (bladder) and respiratory system (lung) provide a preliminary test of the applicability of the proposed framework to multiple tumor indications. We separately reported the prognostic accuracy of the framework by performing cross-validation on both data sets. Overall, the proposed framework achieves state-of-the-art performance in OS prognosis on the NLST data by outperforming previously published approaches and attains statistically significant results for the bladder cohort. We further extended our analysis to evaluate the prognostic significance of different clinical and pathological characteristics through univariable and multivariate analysis. Lastly, to explore the explainability of our results (the ability to present findings so human experts can understand the cause of a decision), we obtain WSI density maps to investigate regions of TME that may have predictive features, and which correlate with pathological interpretation to better understand the disease progression. The WSI density maps highlight, without any prior assumptions or annotations, crucial risk areas that also correlate with pathologist interpretation.

## Results

### Study design

The proposed model was developed and evaluated on two independent cohorts of H&E stained WSIs from lung and bladder carcinoma. The first cohort was a subset (collected from a total of 53,454 participants) of the publicly available data set from the National Lung Screening Trial (NLST)^24^ and the second was an in-house cohort of urothelial cells bladder carcinoma. To demonstrate the robustness of the proposed framework, we separately performed *k*-fold cross-validation for both tumor indications. For each fold, we randomly split the data at the patient level into training (70%), validation (10%), and test (20%) sets. We used the training data to learn the optimal weights for the deep learning model and validation data to monitor the performance of the model and hyper-parameter tuning. The test data set was then used to report the performance and to estimate the generalizability of the model. Overall survival (OS) was used as the prognostic endpoint for this study.

#### Bladder carcinoma

A total of 198 cases of urinary bladder carcinoma were available for this study. The tissue slides were scanned with an Aperio ScanScope whole-slide scanner (Leica Bio-systems Imaging, Inc., Illinois, USA) at ×20 magnification with a microscopic resolution of 0.49 mm/pixel. The clinical data contained histological grade, along with other parameters including gender, ethnicity, age, days to progression-free, and overall survival. A detailed description of this data set is presented in Table 1 (right). For training, we randomly sample 500 regions-of-interest (ROI) each of size 224 × 224 × 3 at ×5 magnification from each WSI using OpenSlide. ROIs selected at higher magnifications may offer a detailed representation of tissue at cell levels, but they lack the spatial information which was previously shown to be effective for natural and histology images^25^. In total, we extracted 99,000 overlapping ROIs (with a maximum stride of 50%) after removing regions not containing tissue using Otsu thresholding based tissue segmentation. Further, we randomly split the data into 4-folds, and we selected 3-folds of the data for training and the remaining 1-fold for testing. We then performed cross-validation by switching the training and test data sets, ensuring every patient’s WSI is used once in the test set.

**Table 1:** A concise summary of clinical and histopathological characteristics for lung (left) and bladder (right) data sets.

Patient characteristics of Lung data set		Patient characteristics of Bladder data set	
Patient characteristics	Summary	Patient characteristics	Summary
Number of patients	*L* = 410	Number of patients	*B* = 198
Total whole-slide images	1122	Total whole-slide images	198
Age	63.8 ± 5.2	Age	73.8 ± 10.6
Gender (Male/female)	60.7%/39.37%	Gender (Male/female)	73.7%/26.3%
Stage		Stage	
Stage I	273	Stage I	0
Stage II	15	Stage II	152
Stage III	63	Stage III	34
Stage IV	18	Stage IV	12
Can not be assessed	28	Can not be assessed	0
Grade		Grade	
Well Differentiated (G1)	50	Grade 1	2
Moderately Differentiated (G2)	156	Grade 2	5
Poorly Differentiated (G3)	132	Grade 3	188
Undifferentiated (G4)	9		
Not known	31	Not known	–
TNM staging		TNM staging	
TX, NX, MX (cannot be measured)	31, 24, 21	TX, NX, MX (cannot be measured)	1,153, 196
T1, N0	301, 297, 369	T1, N0	0, 36
T2, N1, M1	47, 18, 20	T2, N1, M1	153, 4, 1
T3, N2	19, 52	T3, N2	33, 3
T4, N3	12, 19	T4, N3	11, 0
Censored patients (%)	29.75	Censored patients (%)	59.09
Median follow-up time	6.59 y	Median follow-up time	3.67 y
*K*-fold cross validation	*L*_*k* _= 5	*K*-fold cross validation	*B*_*k* _= 4
Number of training image patches	205, 000	Number of training image patches	99, 000

#### Lung carcinoma

The lung data set consisted of 1122 WSIs derived from the formalin-fixed paraffin-embedded (FFPE) tissue specimens of 410 patients selected from the NLST data set. All WSIs were scanned at ×40 magnification with a microscopic resolution of 0.25 mm/pixel using an Aperio ScanScope. Similar to the experiments with the urothelial bladder data, we performed 5-fold cross validation to evaluate the performance of the model. For training, we randomly selected a total of 205,000 image ROIs each of size 224 × 224 × 3 at 5 × magnification. Table 1 (left) represents a list of clinical and demographics features included in this study. The NLST H&E stained images were sampled from blocks of lung tumor tissues that were preserved by pathology labs during diagnosis and treatment of the disease.

### Prognostic evaluation for bladder and lung carcinoma

To evaluate the performance of weakly supervised survival-convolutional neural network (WSS-CNN), we separately computed the prognostic accuracy for each tumor indication using *k*-fold cross-validation. Overall, we assessed the efficacy of the proposed WSS-CNN on >1300 WSIs with their follow-up clinical and demographics data. Both data sets comprised patients with heterogeneous characteristics in terms of variability among histological grade (including well-differentiated, moderately differentiated, and poorly differentiated cases), cancer stage (from I to IV), and TNM staging, as shown in Table 1. Overall survival is generally defined as the duration between the time from either diagnosis or start of treatment and the time to event (death in our case). In this work, for both lung and bladder data sets, the start time was the treatment time. We selected overall survival to report our results in this study because it is widely recognized as a reliable endpoint in most oncology clinical trial studies. As a pre-processing step, we transformed the survival times from days to months for all the follow-up experiments.

**Fig. 1:**
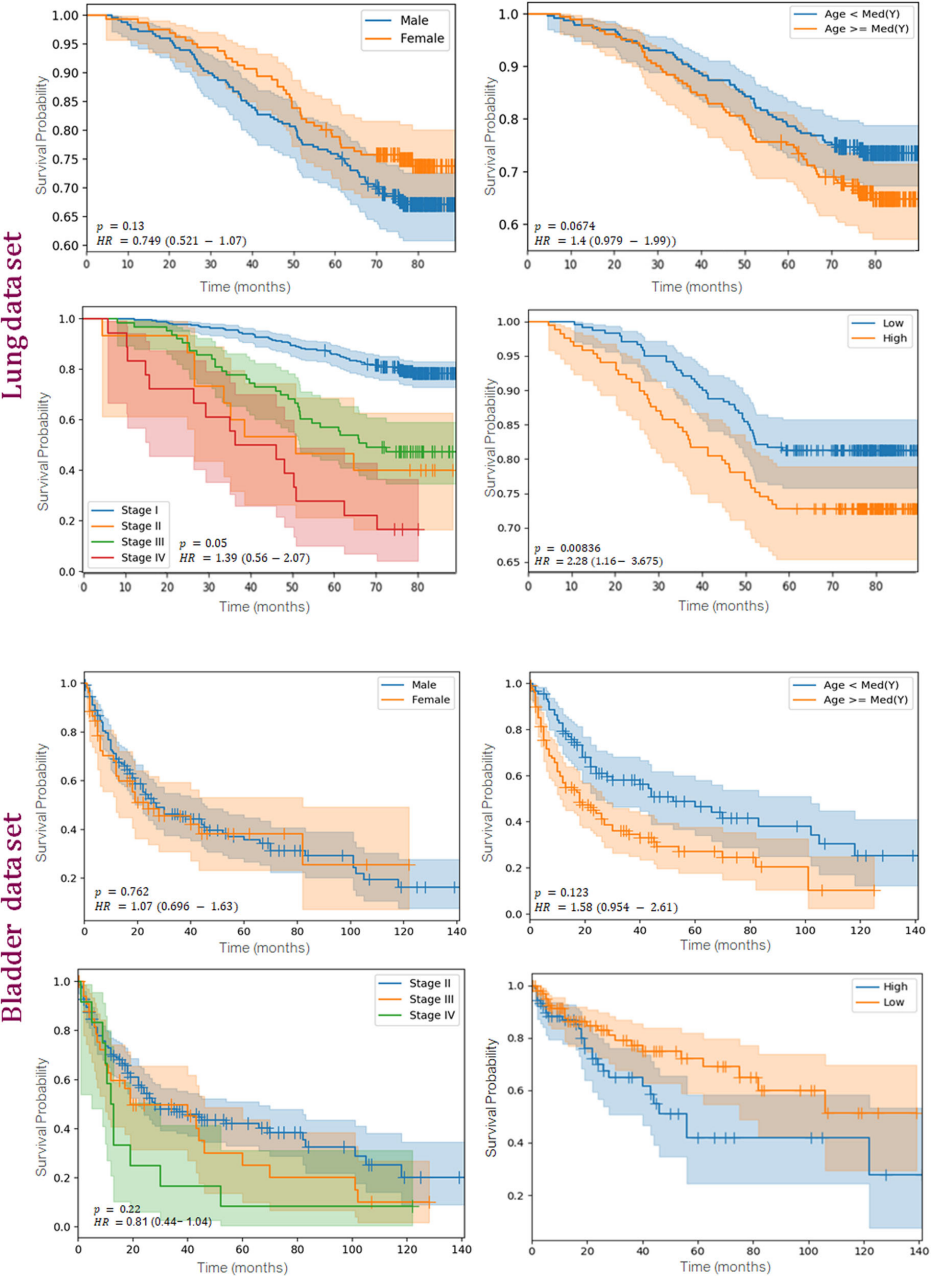
Univariable Kaplan-Meier survival curves for clinical characteristics including gender, age, and cancer stage along with the proposed WSS-CNN. The top two rows show results from lung NLST data and the bottom two rows belong to bladder data. Risk categories (low, high) were identified by using median threshold for WSS-CNN predicted risks.

The reported analysis has two main components (a) evaluate the prognostic ability of the proposed WSS-CNN performing univariable and multivariable Cox regression analysis, along with computing concordance-index (Table 2) (b) Kaplan–Meier survival analysis (Fig. 1) to inspect overall survival difference in high/low stratified groups as compared to standard clinical and demographics features. The hazard ratios in univariable and multivariable analysis were computed using the Mantel-Haenszel method. We used two-sided *p* values and *p* < 0.05 was considered to be statistically significant. In addition, we also performed comparative analysis to demonstrate the prognostic efficacy of the WSS-CNN as compared to different state-of-the-art baseline models (Table 3).

**Table 2:** Univariable and multivariable analysis for overall survival on the test data sets.

Bladder quantitative results: univariable and multivariable analysis for overall survival					
Univariable				Multivariable	
Variable	Hazard ratio	*p* value	c-index	Hazard ratio	*p* value
Age	1.58 (0.954–2.61)	0.123	0.592	1.03 (1.01–1.24)	0.127
Gender	1.07 (0.696–1.63)	0.762	0.533	1.85 (0.69–3.89)	0.517
Tumor purity	1.55 (1.09–1.84)	0.13	0.587	1.92 (1.18–3.03)	0.094
Stage (II vs III & IV)	1.51 (0.9–2.53)	0.22	0.521	2.34 (1.09–5.14)	0.212
WSS-CNN (high/low risk)	1.96 (1.23–2.947)	0.0179	0.612	2.27 (1.72–3.89)	0.0012

Lung quantitative results: Univariable and multivariable analysis for overall survival					
Univariable				Multivariable	
Variable	Hazard ratio	*p* value	c-index	Hazard ratio	*p* value
Age	1.4 (0.979 – 1.99)	0.0674	0.657	1.62 (1.05 - 2.03)	0.004
Gender	0.74 (0.521– 1.07)	0.13	0.633	0.71 (0.44 - 1.16)	0.194
Smoking status	1.39 (0.79 – 2.17)	0.09	0.576	1.95 (1.59–2.54)	0.212
Stage (II vs III & IV)	1.58 (0.66 – 3.78)	0.01	0.642	2.19 (1.12–3.28)	0.118
WSS-CNN (high/low risk)	2.28 (1.16- 3.675)	0.00836	0.7033	2.93 (1.61–4.53)	0.00457

**Table 3:** Comparative analysis of different state of the art baseline models using concordance index value on both data set.

Comparative analysis for bladder and lung data set		
Method	Bladder c-index	Lung c-index
WSS-CNN AlexNet (Krizhevsky et al.^57^)	0.5713	0.623
WSS-CNN VGG (Simonyan and Zisserman)	0.5419	0.653
WSS-CNN ResNet-18 (He et al.)	**0.6125**	**0.7033**
WSS-CNN DenseNet-121 (Huang et al.)	0.538	0.666
DeepSurv (Katzman et al.^63^)	–	0.602
DeepConvSurv (Zhu et al.)	–	0.629

(where bold values represent the best performance)

Generally, the prognostic role of age, gender, and molecular subtyping features are well explored and provide insights to support clinical decisions. Tumor purity or the proportion of tumor cells in the whole tissue section is another independent factor that is routinely determined by pathologists and is associated with histological grades, and disease prognosis^26,27^. Overall, the WSS-CNN model outperformed other standard clinical factors and achieve the c-index of 0.7033 (*p*-value: 0.00836) for lung and 0.6125 (*p*-value: 0.0179) for the bladder, as shown in Table 2. Conventionally, the Cox proportional hazards model forms a linear combination of covariates (e.g. age, gender, tumor purity, etc) to estimate the patient’s risk. Due to the heterogeneous appearance of TME with complex interactions of tissue components, generally non-linear risk functions are more suitable for estimating survival. It is encouraging that a weakly supervised approach shows prognostic significance over cancer stage (bladder: c-index:0.521 and *p*-value: 0.195) and tumor purity (bladder: c-index: 0.587 and *p*-value: 0.13) (Table 2, top). Intuitively, younger patients may experience a favorable prognosis than older patients and our results show a comparable prognostic significance of age for lung patients (with c-index: 0.657 and *p*-value: 0.0674). Similar to the bladder data set, the c-index for clinical and demographics parameters are relatively low as compared to the WSS-CNN for lung data set. Regarding comparative study, we investigated the prognostic significance of different baseline models (including AlexNet^28^, VGG^29^, ResNet^30^, and Dense-Net^31^) as shown in Table 3. The residual connection in intermediate convolution layers of ResNet enables the gradient to flow smoothly during training and eventually assists in achieving better prognostic accuracy. Besides, we also evaluated the impact of the visual attention mechanism by separately training the WSS-CNN framework with ResNet-18 for each tumor indication. Excluding the visual attention mechanism from WSS-CNN marginally drops the overall performance to 0.6863 (for lung) and 0.5911 (for bladder).

We then performed Kaplan-Meier analysis to further investigate the prognostic significance of WSS-CNN and to observe the differences in overall survival among patients stratified as high and low risk categories using the log-rank test, as shown in Fig. 1. For WSS-CNN predictions, the cut-off values used for risk group stratification (low/high progressors) were based on the median of risk scores after performing cross-validation on the entire cohort. Median of predicted risk scores was used as a threshold to stratify the given cohort (lung or bladder) in high and low sub-groups. We perform univariable Cox regression analysis to examine the independent prognostic significance of WSS-CNN and other clinical and histology features including age, gender, tumor purity, histology grade, and smoking status. In addition, we conduct multivariable Cox regression analysis to investigate the prognostic ability of WSS-CNN risk along with the impact of patients’ clinical and histopathological characteristics. We also report hazard ratios for both univariable and multivariable analyses.

For univariable analysis of bladder carcinoma, the WSS-CNN model was prognostically significant (HR:1.967 (1.23–2.947), *p*-value: 0.0179) whereas disease stage, age, and gender were not. Tumor purity for bladder patients attains a relatively higher c-index (0.587) as compared to other clinical parameters but the overall results were not statistically significant (Table 2). In multivariable analysis, the WSS-CNN shows independently prognostic (HR:2.27 (1.72–3.89), *p*-value: 0.0012) for overall survival using other patient characteristics. In lung univariable analysis, the WSS-CNN shows prognostic relevance (HR:2.28(1.72–3.675), *p*-value: 0.00836) in contrast to standard clinical and histology features. Based on Kaplan–Meier survival curves and hazard ratio, gender, and smoking status show the least prognostic relevance for estimating overall survival. For lung multivariable analysis, the WSS-CNN (HR:2.93(1.61–4.53), *p*-value: 0.00457) as age (HR:1.62(1.05–2.03), *p*-value: 0.0048) shows prognostic of overall survival. Normally, elder patients diagnosed with lung cancer may observe poor disease outcome as compared to young patients.

### Pathological interpretation of tumor micro-environment predicted regions

**Fig. 2:**
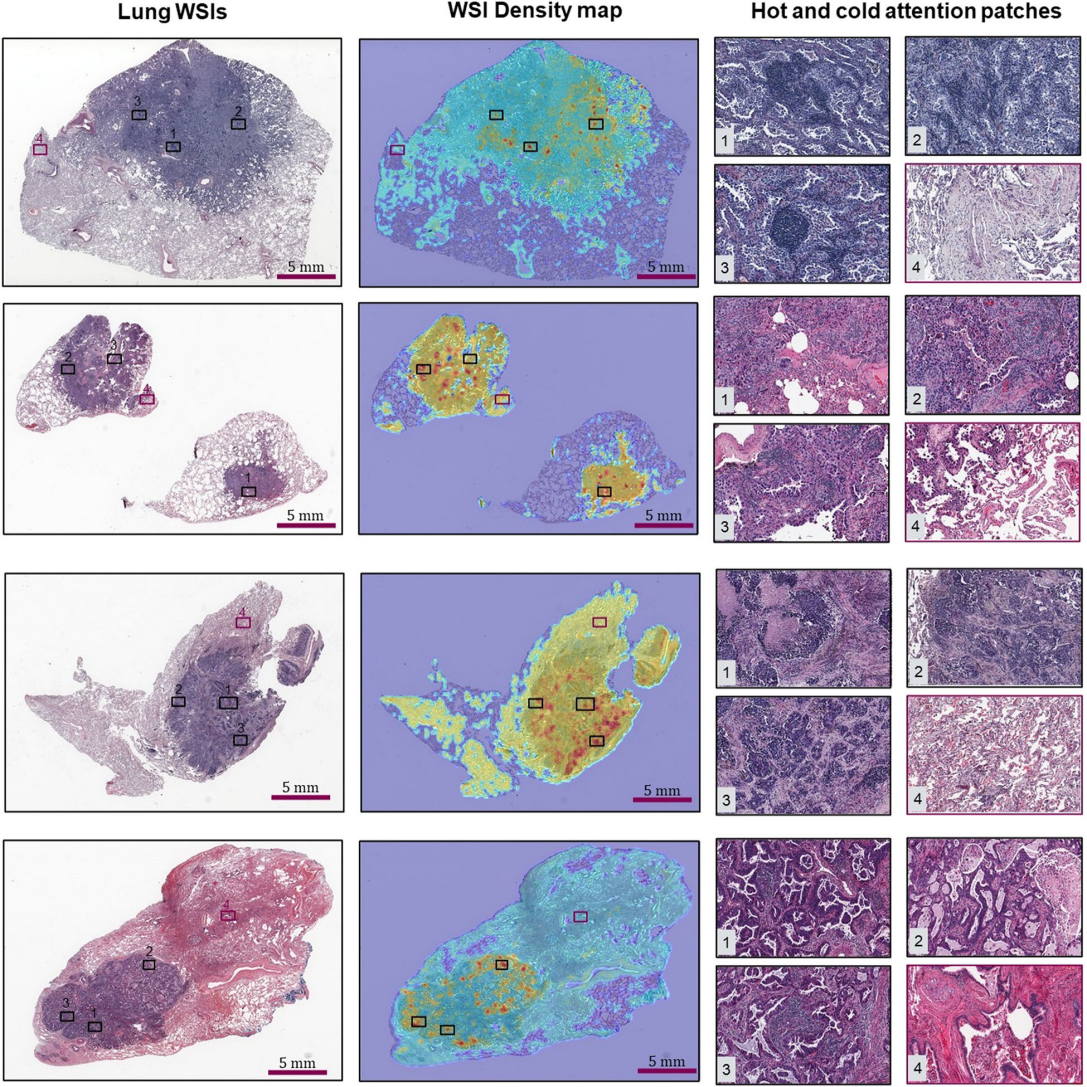
Density risk maps of whole-slide images processed by WSS-CNN from the bladder data set. The WSS-CNN analyzes all the tissue regions within a given whole-slide image to generate a risk map that WSS-CNN associates with different histologic patterns. The red color indicates relatively higher risk, and blue indicates lower risk.

**Fig. 3:**
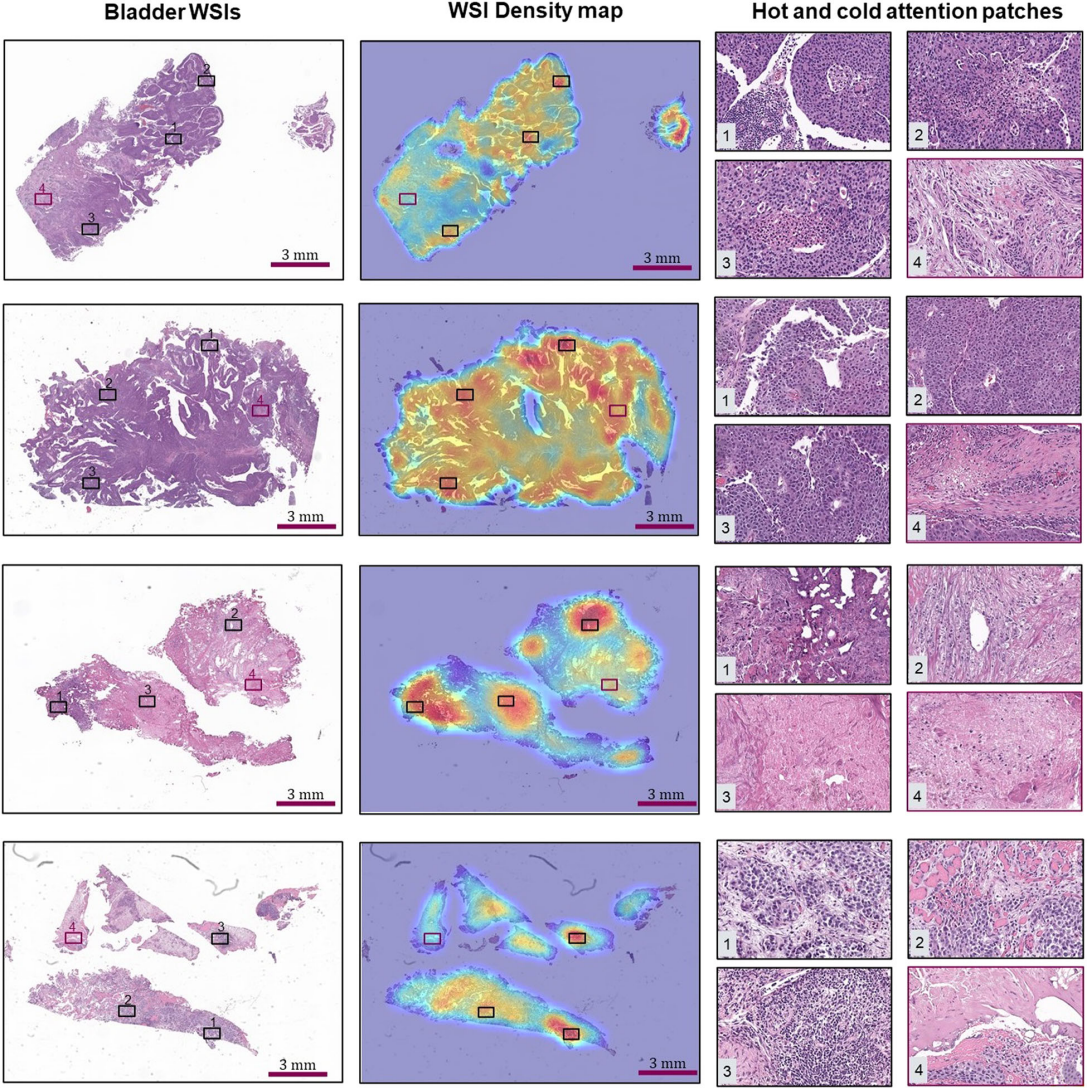
Density risk maps of whole-slide images produced by the WSS-CNN framework from the lung NLST data set. Similar to bladder data, the red color in density map indicates relatively higher risk, and blue indicates lower risk regions on the WSI level.

In order to promote the adoption of AI-based approaches in routine clinical practice, it is crucial to enable these approaches to produce interpretable outcomes which may assist experts in better understanding the relationship between the ML features and disease pathomechanism. In this regard, we separately explore the pathological interpretations of tumor micro-environment predicted regions for both data sets using risk density map visualizations. We overlaid transparent risk density maps on H&E stained WSIs which enable pathologists to correlate the model predictions with the underlying histology of the disease. Density maps were generated using a trained WSS-CNN model to predict the risk for each ROI in a whole-slide image. The ROI predicted risks were then aggregated to WSI-level, followed by a color map to overlay on WSI, where red and blue indicate higher and low WSS-CNN risk regions, respectively. A selection of risk heat maps from multiple patients are presented in Fig. 2 (bladder) and Fig. 3 (lung), with ROIs showing how WSS-CNN’s predictions correlated with important pathological phenomena.

Figure 2 presents the WSI-level risk density maps for a set of four patients from the bladder data set. The risk maps show a combination of multifocal and diffuse TME delineated regions signifying their associated risk predicted by WSS-CNN. The top two rows manifest multifocal patterns and both patients belong to slow progressors (better prognosis) with tumor stage II, age: 68 and 44, and primarily diagnosed as transitional cell carcinoma and papillary urothelial carcinoma, respectively. The WSS-CNN density maps mostly exhibit cancerous tissue areas with pleomorphic regions containing large hyperchromatic nuclei and moderate mitotic activity as high-risk. The risk maps also identify regions containing high cellularity with heterogeneity in nuclei appearance and size (pleomorphism), interestingly, these features are previously reported as a significant prognostic factor for urothelial carcinoma^32,33^. The density maps in the bottom two rows show a number of diffuse patterns dispersed over different regions of TME and both these patients experienced poor prognosis (fast progressors) with cancer stage II, age 42 and 66, and primarily diagnosed as urothelial carcinoma. Similar to previous examples, the density maps broadly identify high-risk regions within the malignant tissue containing complex irregularly arranged nest’s structure, necrotic tumor regions^34–36^, and angiogenesis^37,38^ showing abnormal microvascular structures. It is noteworthy to mention that most of these features are previously reported as tumor-promoting factors and are widely associated with poor prognosis.

The attained risk maps for four patients from the lung data set are shown in Fig. 3. Similar to the bladder data, the lung WSI-level risk map links the association of WSS-CNN predicted regions with their pathologic interpretation. One of the most interesting aspects of lung density maps is the well-delineated localization of tumor-enrich areas (shown as either yellow or teal color) without using any expert’s ground-truth information. Furthermore, most of the risk maps demonstrate multifocal patterns of high-risk areas within tumor regions, highlighting the significance of malignant regions for lung prognosis. For slow-progressors (top two rows), the high-risk regions mostly contain sites of tumor-infiltrating lymphocytes (TIL), inflamed stromal regions, and tertiary lymphoid structures (TLS). A number of previous studies have identified these as an independent prognostic factor and the presence of inflamed stromal regions is linked with prolonged survival. The WSI risk density features for fast-progressors (in the bottom two rows) show TIL regions with adjacent tumor and lymphocytes interaction, localization of invasive edge margin, and necrotic regions^39–41^. Previously, along with other patterns, tumor necrosis in the lung was previously reported to be an adverse prognosis risk factor^42–44^. These risk maps with multifocal patterns precisely localize high-risk histology patterns which could potentially identify novel spatial signatures relevant to disease pathomechanisms.

## Discussion

The science of therapeutic evaluation in clinical trials is on the verge of imminent transformation due to the availability of large-scale digitized data sets, emerging compute power, remarkable developments in artificial intelligence and machine-learning algorithms, and the escalating acceptability of software as a medical device^45^. Recently, enormous efforts have been made to improve the interpretability, reliability, and efficacy, of the clinical trial studies^46^. One of the emerging directions to improve the reliability of clinical trials is to precisely predict the occurrence of relevant events such as death, disease progression, the occurrence of severe adverse events such as stroke, infection, or organ failure. This work aimed at presenting a weakly supervised visual attention-based automated approach to estimate prognosis from H&E stained histology WSIs of lung and bladder carcinoma.

Experimental results attained through systematic evaluation on two independent cohorts demonstrated the efficacy of deep learning-based WSS-CNN on H&E stained histology images. Moreover, our presented results have several interesting aspects, (a) it overcome the need for manual annotations which makes this approach more applicable to large-scale studies, where collecting precise annotations of scanned WSIs would be a laborious and time-consuming task, prone to observer variability. To the best of our knowledge, this is the first study to show the utility of weakly supervised learning with visual attention to predict patient survival from H&E stained WSIs, (b) there is an increasing interest to utilize weakly supervised learning (WSL) for the prediction of relevant clinical outcomes as it is less time-consuming and inexpensive relative to supervised methods. Another approach^47^ based on WSL employs a customized loss function to transform survival prediction as a classification problem by splitting the survival time into four discrete bins based on censored information. The performance of such approaches may rely on the number of discrete bins which may vary among different data sets. Unlike other approaches, the proposed WSS-CNN leverages weak supervision among randomly sampled H&E stained image patches and utilizes visual attention to emphasize only prognostically relevant regions of an ROI (c) for better interpretation of the AI-based results and to get more insights into prognostically relevant TME spatial signatures, we analyze the risk maps of the WSS-CNN to correlate prognostic features with pathological interpretations. This analysis may assist in developing a better understanding of disease pathomechanism, (d) we perform *k*-fold cross-validation, to estimate the model generalizability on unseen splits of the data set. This ensures that every patient’s WSI is used exactly once in the test set, and *k* − 1 times during training (e) we perform univariable and multivariable Cox regression analysis to investigate the prognostic significance of H&E image along with other clinical and demographics variables (e.g. cancer stage, histology grade, gender, age, etc).

This study investigates the significance of deep learning and H&E stained whole slide images to predict overall survival for two heterogeneous tumor indications including lung and bladder urothelial carcinoma. Lung cancer is the most common and prevalent form of cancer worldwide, accounting for nearly 18% of the cancer-related mortalities^48^. On the other hand, bladder cancer is the 10th most common form of cancer, accounting for nearly 2.1% of total cancer-related deaths each year with a growing number of cases, especially in western populations^49^. Previous work-related to automated survival analysis often trains customized neural work but differed from these approaches, we enable SOTA models to predict survival, we observed that ResNet-18^30^ produces a better concordance index as compared to other SOTA models. In multivariable Cox regression analysis, WSS-CNN median risk was independently significant for overall survival, along with a set of covariates including histology grade and age for lung cohort, as also demonstrated in Kaplan–Meier analysis (Fig. 1). In most clinical trial studies, overall survival is one of the primary events of interest and is our main intent of using it for reporting our analysis.

AI model interpretability is an indispensable aspect of machine learning especially when applied to clinical data. Interpretable AI models may deftly secure the confidence of clinicians and patients in adopting cutting-edge digital health solutions. The risk maps (shown in Figs. 2 and 3) are representing focal or diffuse patterns highlighting crucial TME components, such as necrosis, pleomorphic regions with hyperchromatic large nuclei, and angiogenesis for bladder cohort and tumor-infiltrating lymphocytes, inflamed stromal regions, necrosis, and tertiary lymphoid structures for lung data set. Tumor necrotic regions, as identified by the WSS-CNN (Fig. 2, 3rd row and Fig. 3, 2nd row)) have previously reported as an independent prognostic factor linked to poor disease outcome^34^. This kind of analysis would not only improve the interpretability of deep learning models but also transform H&E stained gigapixels images into a sequence of interpretable information. Moreover, it may also provide insights into disease patterns associated with poor disease outcomes and guide experts in unraveling the disease heterogeneity.

This study presents an emerging deep learning tool that may assist in optimizing clinical trial design but it also comes with some limitations. The model is trained on random samples extracted from WSIs; automated, semi-automated, or some curriculum-based learning (e.g. how many tissue regions to be selected from each slide, etc) could be explored for the selection of ROIs to train the model^50^. Further extension of the WSS-CNN framework may entail validating the performance of the model on larger cohorts, preferably containing a uniform distribution among different stages of the disease. Another potential extension would be to predict survival using the combination of different data modalities, e.g imaging-to-imaging data (by using radiology data as supplementary information along with H&E data) or imaging-to-tabular data by exploring genomics or other patient patients’ characteristics along with imaging data^51^. Different scanning protocols introduce domain shifts which may pose challenges for automated algorithms. To train a scanner-agnostic computational pathology system, one solution (and a future extension of this work) could be to diversify the training data by collecting data from multiple scanners. For our Kaplan-Meier analysis, the cut-off values used for risk group stratification (high/low progressors) were based on the median of feature vectors, it is worthwhile to explore more optimal ways of finding the cut-off on a larger cohort. We prioritize cancer stages from both data sets for KM analysis mainly due to skewed distribution of cases across histology grades (especially for bladder data). For univariable and multivariable analysis, we combined cases across cancer grade (G1 with G2 and G3 with G4) based on data distribution and generally, G3 and G4 are often categorized as high-grade tumors with abnormal cells that are more aggressive as compared to G1 and G2. For future analysis on larger cohorts containing uniform distribution, it may be worth exploring different combinations of tumor grades and cancer stages. The interpretation of risk maps was based on visual evaluation, and a comprehensive study to investigate risk maps more objectively would assist in finding novel TME digital biomarkers associated with different sub-groups (e.g. high/low). Lastly, there was 1 patient in the lung data and around 15 patients in the bladder data that underwent neoadjuvant treatment and such factors may also impact OS for those patient. It is an important point and warranting further investigation on larger cohorts where there is higher proportion of patients with different pre-treatment history. In real world data sets, especially when tissue slides were gathered from nonserial sections, it is apparent that all the tissue slides might not be equally informative for predicting clinical outcomes. In order to select the most informative tissues slide(s), one may need an intermediate process to either manually or automatically select the best possible slide(s). We have presented some preliminary analysis in this regard, as provided in the Supplementary Information section 1.

Prospective validation of the WSS-CNN framework in clinical trial studies would be crucial prior to utilizing it as an independent clinical tool. One potential application could be to use this in clinical trial settings to stratify participants as fast (high risk) and slow progressors (low risk) based on their disease outcome. This would enable clinicians to select relevant participants for a multi-arm clinical trial study and to better access the treatment response, drug efficacy, and safety within different sub-groups. We believe the proposed framework carries the potential to be applied not only to other tumor indications but also to other medical imaging modalities. This work would likely pave the way for the development of other AI-based imaging approaches in predicting relevant clinical outcomes and optimizing the clinical trial design for precision medicine.

## Methods

Each WSI is a gigapixel image (10^10^ pixels) with an average spatial dimensions of 50, 000 × 80,000 (15mm × 25mm) and an uncompressed version of a WSI may require 56GB^52^ of storage at ×40 magnification. Analyzing the entire WSI at once is a computationally expensive task and therefore we split each WSI into manageable ROIs. The model was trained on randomly extracted image patches from WSIs using WSI-level ground-truth survival information provided for each patient. This transforms it into a weakly supervised learning problem where we only have the patient-level prognosis information and we do not have any precisely marked regions from pathologists.

### Ethics statement

The lung data from NLST was collected after institutional review board approval at each contributing center and the National Cancer Institute. The informed written consent was obtained from all participants involved in the NLST^53^. Regarding bladder commercial samples, AstraZeneca has a governance framework and processes in place to ensure that commercial sources have appropriate patient consent and ethical approval in place for collection of the samples for research purposes, including use by for-profit companies. The AstraZeneca Biobank in the UK is licensed by the Human Tissue Authority (Licence No. 12109) and has National Research Ethics Service Committee (NREC) approval as a Research Tissue Bank (RTB) (REC No 17/NW/0207), which covers the use of the samples for this project.

### Disease prognosis without precisely annotated ground-truth (or ROIs)

Weak supervision is a variant of supervised learning from the machine learning paradigm where the model is trained on partially or weakly annotated data^54^. Collecting precisely annotated ROIs for a large number of WSIs is often expensive, time-consuming and a significant obstacle for real-world data sets. Recognizing this obstacle in training fully supervised learning algorithms, we propose a weakly supervised survival-convolutional neural network (WSS-CNN) framework incorporating a visual attention mechanism to overcome the dependency of learning algorithms on annotated regions.

**Fig. 4:**
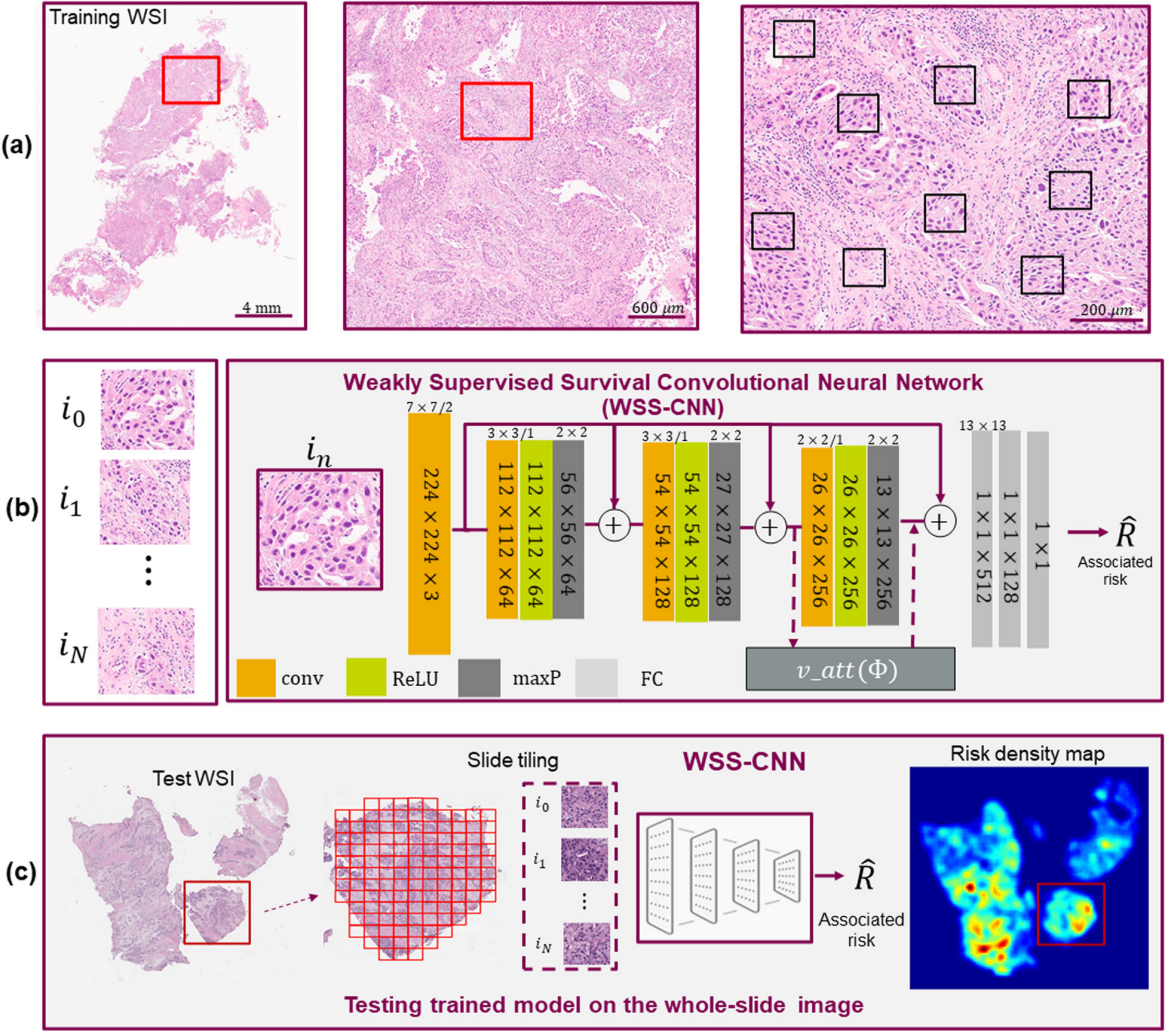
Overview of the proposed prognosis pipeline for analyzing histology H&E stained whole slide images (WSIs). **a** shows an example of ROI (left image) from a WSI from which multiple patches were randomly selected during training. We sample *N* image patches *i*_*N*_ where *N* = {1, . . . , *n*}, each of size 224 × 224 × 3 from all WSIs of a patient. Each *i*_*n*_ was further transformed by data augmentations to generate synthetic copies which were used during training the model. **b** Extracted image patches (regions of interest) *i*_*N*_ were then used to train a weakly supervised survival convolutional neural network (WSS-CNN) to predict overall survival. The WSS-CNN comprised a combination of fully connected layers and convolutional layers which were followed by max-pooling and non-linearity. Visual attention mechanism and residual connections using element-wise summation are also shown in the illustration. **c** While testing, we run inference on the WSI level and to evaluate the performance of the model for an unseen patient. Each image patch from the tissue regions *i*_*n*_ was analyzed by the WSS-CNN to predict the associated risk.

Estimating prognosis (or time-to-event) from histology images is considered a more sophisticated problem than a conventional regression task, largely due to censored observations, in case, one or more patients have not experienced the relevant outcome (death, relapse, etc) during the duration of the study or if the patient’s outcome is unavailable which in general makes it impractical to track the survival status. To optimize the WSS-CNN learnable weights in the presence of censored data, we employed the Cox proportional hazards model^55,56^ as the loss function which calculates the negative partial likelihood to predict patients’ outcome. A detailed illustration of the proposed WSS-CNN model is shown in Fig. 4. The ROIs used for training the WSS-CNN contained a heterogeneous representation of the tumor micro-environment including image patches from malignant and normal epithelial, stromal, and lymphocytic regions, etc. The convolutional neural network in WSS-CNN serves as a non-linear function that maps a given ROI patch into high-dimensional prognostic features which then use to predict the associated risk. The attention mechanism is a combination of spatial and channel attention which enables the WSS-CNN to refine the activation maps by emphasizing the prognostically relevant regions. It is worth noting that our attention mechanism does not include any additional training parameters hence the complexity of the models remains the same. To aggregate the results on the WSI level, during inference, we process all the tissue regions of a given WSI.

### Model training and network architecture

Previously, several automated survival analysis approaches were presented either using patient’s demographics (or structural prognosis features) or using hand-crafted features from H&E stained images by training a shallow machine learning model^18–20^. The hand-crafted features are generally data-specific and sensitive to data variations. There also exist some approaches that utilize deep convolutional neural networks but most of these approaches proposed customized convolutional neural networks which are often tailored to solve a specific task and need proper architecture calibrations (number of hidden layers, neurons per layer, etc). In this work, we enable the state-of-the-art (SOTA) baseline models (or representative models) for predicting survival. More specifically, the SOTA models we used are ResNet-18^30^, VGG-11bn (batch normalization)^29^, DenseNet^31^, and AlexNet^57^.

Given a H&E stained WSI *I*, the objective of WSS-CNN is to learn prognostically relevant discriminative features and to predict time-to-event by analyzing a set of image ROIs *i*^*n*^ ∈ *R*^*H*×*W*×*D*^, where $I={\{({i}^{n})\}}_{n = 1}^{N}$. The schematic diagram of the proposed model is shown in Figure 4(b). The WSS-CNN has two main components including a) a deep CNN baseline model and b) a visual attention mechanism (which is described in the following section). Here, the baseline model acts as a non-linear mapping function *f*(*i*^*n*^) → ***x***^*n*^, where ${{{{\boldsymbol{x}}}}}^{n}=({x}_{1}^{n},...,{x}_{p}^{n})$ is the feature vector of *p* dimensions. The CNN model is an altered version of residual CNN (ResNet^30^) that contains multiple residual connections to reroute the low-level features and combine them with intermediate and high dimensional feature representations. The fully connected layers contain non-linear weighted combinations of convolutional features, which then feed into the final layer that predicts associated risk. The Cox proproportional-hazards model is a commonly used regression model associating prognosis with predictor variables of patients. The hazard represented by Cox proportional model is defined as, $h(t| {{{\boldsymbol{x}}}})={h}_{0}(t)\exp ({\beta }^{T}{{{\boldsymbol{x}}}})$, where *h*_0_ is the baseline hazard, *t* is the survival time, *β* = (*β*_1_, . . . , *β*) denotes the regression parameter vector of dimension *p*. The goal here is to estimate vector *β* by minimizing negative log partial likelihood. The proposed model is trained end-to-end by maximizing the performance over the model parameters. The cost function is the negative log partial likelihood is *l*(*β*) as given in (1),

1 $$l(\beta)=-\mathop{\sum }\limits_{m=1}^{M}{\delta }_{m}\left({\beta }^{T}{x}_{m}-log\mathop{\sum}\limits_{j\in R({t}_{m})}exp({\beta }^{T}{x}_{j})\right)$$

where *M* is the number of patients, *x*_*m*_ represents feature vector for the input image *i*^*n*^, and *δ*_*m*_ indicates if the survival time is censored (*δ*_*m*_ = 0) or observed (*δ*_*m*_ = 1) for patient *m*, *R*(*t*_*m*_) is the set of risk at time *t*_*m*_ for all individuals still under study. Finally, to aggregate the ROI level *i*^*n*^ results to the patient level (or WSIs level), we calculate the central tendency (median) of the ROI risk values. The ROI risks were first sorted and then the central value is obtained, separately for each patient. Computing measures of central tendency also ensures the exclusion of noisy (outliers) and relatively less prognostic regions to predict a more robust patient-level survival.

To improve the generalizability and to reduce overfitting, during training, we extensively performed the data augmentation (generating synthetic copies of the original data) by flipping (along the horizontal or vertical axis), random cropping, rotating (0,90,180,270), and perturbing the color distribution (hue variation) for both cohorts, separately. In addition, we used random erasing which randomly picks a rectangular region from an input image *i*^*n*^ and replaces its pixel values with random values. We used AdaGrad stochastic optimization algorithm to minimize the negative log partial likelihood using backpropagation. The initial learning rate was selected as 0.0001 with an exponential reduction of 0.97 and with an adjusted momentum of 0.95. All the learnable parameters of the WSS-CNN were initialized as Gaussian random numbers with 0 mean and 10^−2^ standard deviation. The data normalization was performed using the mean and standard deviation of the ImageNet dataset.

### Visual attention

**Fig. 5:**
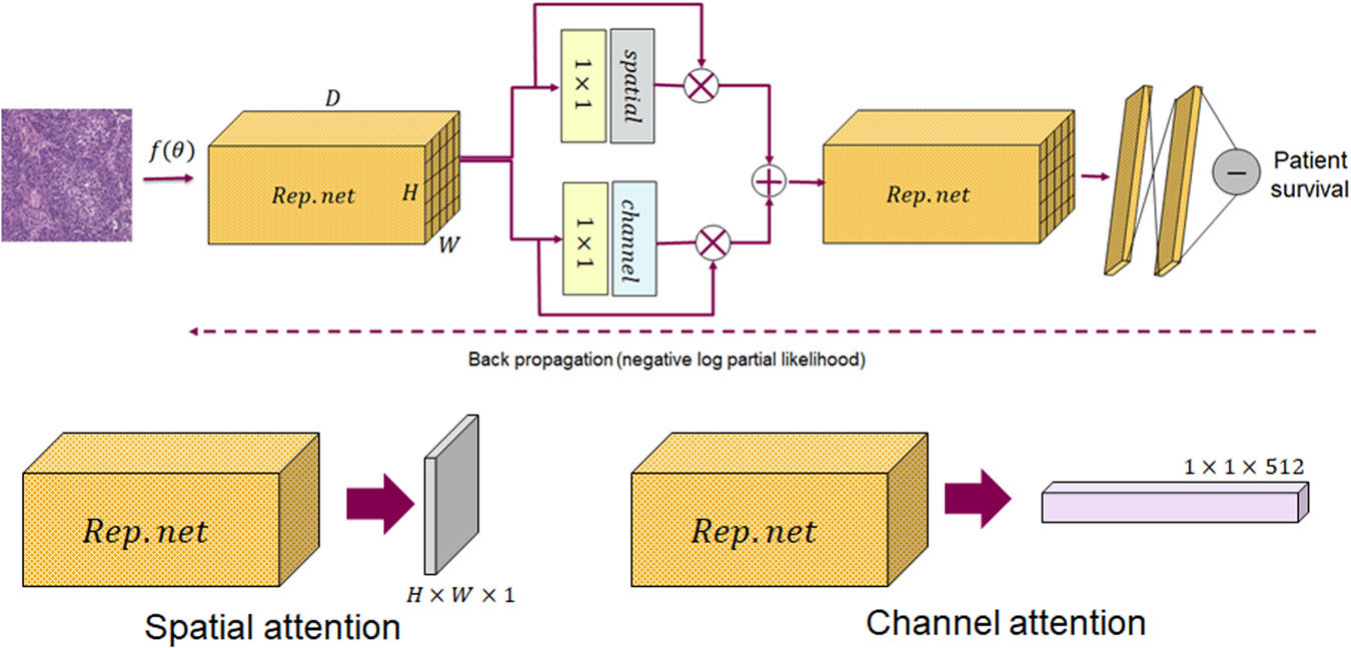
The proposed attention mechanism for analyzing histology images. The module has two main components **a)** spatial and **b)** channel attention which refine the features from an intermediate convolutional layer, as separately shown in the bottom row. Rep.net means representational CNN that process each *i*_*n*_ and transform them to the features maps which were then used for the attention mechanism.

One of the main aspects of human visual perception is that we do not usually process all the information from a given scene but instead we only put attention to a sequence of regions to understand the entire visual scenario. Similarly, in a routine diagnostic setting, while performing the visual examination of tissue slides, a histopathologist would not equally analyze each component of the tissue to make clinical decisions. Recently, there is an increasing interest in incorporating attention mechanisms to improve the deep convolutional neural network performance, mainly for classification tasks. Here, we employ a soft visual attention mechanism^58–60^ that refines input convolutional features and enables the deep learning model to emphasize the task-related features, rather than learning representations from prognostically irrelevant or background regions. The schematic illustration of the attention module is shown in Fig. 5. For a given intermediate convolutional features **F** ∈ *R*^*H*×*W*×*D*^ of the WSS-CNN, the attention mechanism fuses the spatial (*S*_*att*_(**F**) ∈ *R*^*H*×*W*×1^) and channel-wise *C*_*att*_(**F**) ∈ *R*^1×1×*D*^) features together with input convolutional features. The overall concept of the attention mechanism can briefly be described as in (2) and (3)

2 $${{{{\boldsymbol{F}}}}}_{{C}_{att}}={C}_{att}({{{\boldsymbol{F}}}})\otimes {{{\boldsymbol{F}}}}\,{{\mbox{and}}}\,{{{{\boldsymbol{F}}}}}_{{S}_{att}}={S}_{att}({{{\boldsymbol{F}}}})\otimes {{{\boldsymbol{F}}}}$$

3 $${{{{\boldsymbol{F}}}}}_{{C}_{att}+{S}_{att}}={C}_{att}({{{\boldsymbol{F}}}})\otimes {{{\boldsymbol{F}}}}+{S}_{att}({{{\boldsymbol{F}}}})\otimes {{{\boldsymbol{F}}}}$$

where *C*_*att*_(**F**) and *S*_*att*_(**F**) representing channel and spatial-wise attention, respectively and ⊗ denotes the Hadamard product between intermediate convolutional features and attention maps.

In a CNN model, each channel of a convolutional layer contains a certain feature response. The main intent of channel-wise attention (*C*_*att*_) is to interpret the significance of each feature response by first performing 1 × 1 convolution operation followed by a channel-wise global average pooling which transforms **F** ∈ *R*^*H*×*W*×*D*^ → *R*^1×1×*D*^. After compressing the spatial dimension of the feature maps **F**, we normalized the channel attention using the Sigmoid function. Finally, we compute the Hadamard product between the (*C*_*att*_) and **F** to obtain the combined feature vector representing 3D channel-wise attention maps. The spatial branch of the attention module transforms **F** ∈ *R*^*H*×*W*×*D*^ → *R*^*H*×*W*×1^ to generate a 2D spatial attention map which enables the WSS-CNN model to highlight the spatial features of prognostically relevant regions (and suppress features from non-informative regions). To compute the spatial attention *S*_*att*_(**F**), we first perform 1 × 1 convolution which then followed by an inter-channel global average pooling to aggregate spatial information of a given feature map **F**. The 2D spatial attention map is then normalized using the Sigmoid function. Lastly, to obtain the 3D feature maps, we perform the Hadamard product between the *S*_*att*_ and **F**. After obtaining the channel *C*_*att*_(**F**) and the spatial attention *S*_*att*_(**F**), to generate the final 3D refined feature maps, we perform element-wise summation which offers better gradient propagation as compared to other operations e.g., product, or average/max operations.

### Concordance index

Due to censored patients and skewed survival data distribution, survival analysis can be transformed into a ranking problem so rather than predicting the continuous survival time it can infer as ranking the survival time^61^. Therefore, during training, we compute the prognostic accuracy of the model using Harrell’s concordance index (or c-index), which is a non-parametric global statistics measure to quantify the proportion of correctly ranked survival times by total possible actual observation^62^ and formally defined as in (4)

4 $$CI=\frac{1}{| Q| }\mathop{\sum}\limits_{(m,n)\in Q}I(R({x}_{m}) \,<\, R({x}_{n}))=\frac{1}{| Q| }\mathop{\sum}\limits_{(m,n)\in Q}I({\beta }^{T}{x}_{m} \,<\, {\beta }^{T}{x}_{n})$$

Larger c-index values indicate better predictive ability of the model, with c-index of 1 indicating best concordance whereas 0 the worst concordance, and 0.5 means random concordance. It is worth mentioning that the c-index was computed on the data set level, whereas during training, we only compute c-index for a mini-batch and therefore, it is important the way we sample mini-batches for training the model. We ensure that we get image patches from unique patients for each mini-batch after shuffling the training data set.

### Reporting summary

Further information on research design is available in the Nature Research Reporting Summary linked to this article.

## Supplementary information

REPORTING SUMMARY

Supplementary File

## Data Availability

The NLST data is a publicly available data set and can be requested form the following link, https://www.cancer.gov/types/lung/research/nlst. The bladder data that support the findings of this study may be shared upon reasonable request to the corresponding author (talha.qaiser1{at}astrazeneca.com) without relevant conflicts of interest for non-commercial use and agree not to distribute the data.
