# Supplementary material for: Usability of deep learning and H&E images predict disease outcome-emerging tool to optimize clinical trials
Source: NPJ Precis Oncol. 2022 Jun 15;6:37. doi: 10.1038/s41698-022-00275-7 (PMC9200764; doi:10.1038/s41698-022-00275-7)
Supplement: Supplementary file 2 — Supplementary File [file 41698_2022_275_MOESM2_ESM.pdf]

Supplementary Information

1. Whole-slide Image Level WSS-CNN Risk Analysis

From the clinical deployment perspective, it is important to evaluate the robustness of the risk score produced by the WSS-CNN for each WSI from the same patient. In order to understand the robustness of the WSS-CNN risk scores for each WSI of the same patient, we performed the following analysis. For each data fold of the lung data, we separately computed the median risk for each WSI of each patient across the entire fold. Then we selected patients containing multiple WSIs and computed the minimum score, lower quartile, median, upper quartile, maximum score, and inter-quartile range (IQR) to be presented in box plots. The computed statistics for all the data folds are represented in Fig. S1. and Fig. S2. Each box plot in Fig. S1. and S2 represents a unique patient with computed statistics based on their predicted risk scores for multiple WSIs. The majority of patients with multiple WSIs (around 67.74%) had a standard error of their predicted risk ≤ 3 and exhibit low variability (as in IQR range) despite being individually evaluated on each WSI from the same patient. In each fold, we also observed a few cases exhibiting higher variability among WSIs of the same patients, and such cases might be further benefited from an intermediate automated or manual approach that can select the most informative WSI(s) for each patient. It is worth mentioning that such intermediate approaches need to be precisely calibrated to operate with high sensitivity to mitigate the chances of excluding informative slides during this process.

We believe the proposed approach would pave the way for the further development of other AI-based imaging approaches in predicting relevant clinical outcomes and to design intermediate processes to identify the most informative tissue slides for further optimizing the clinical trial design in precision medicine.

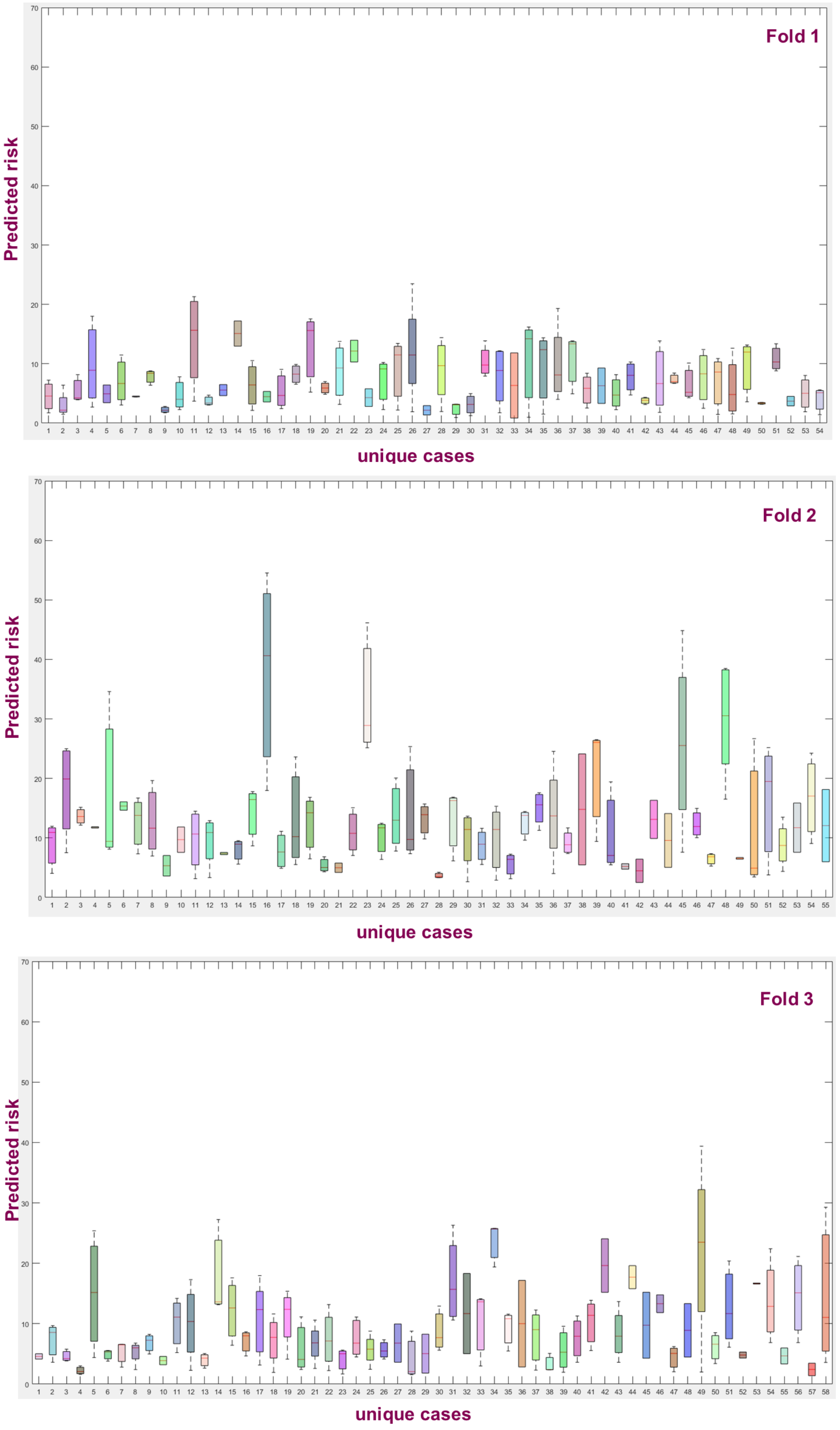

Supplementary Figure 1. The WSS-CNN WSIs level risk prediction analysis for the first three folds of the lung data set. Each box plot presents a unique patient containing multiple WSIs.

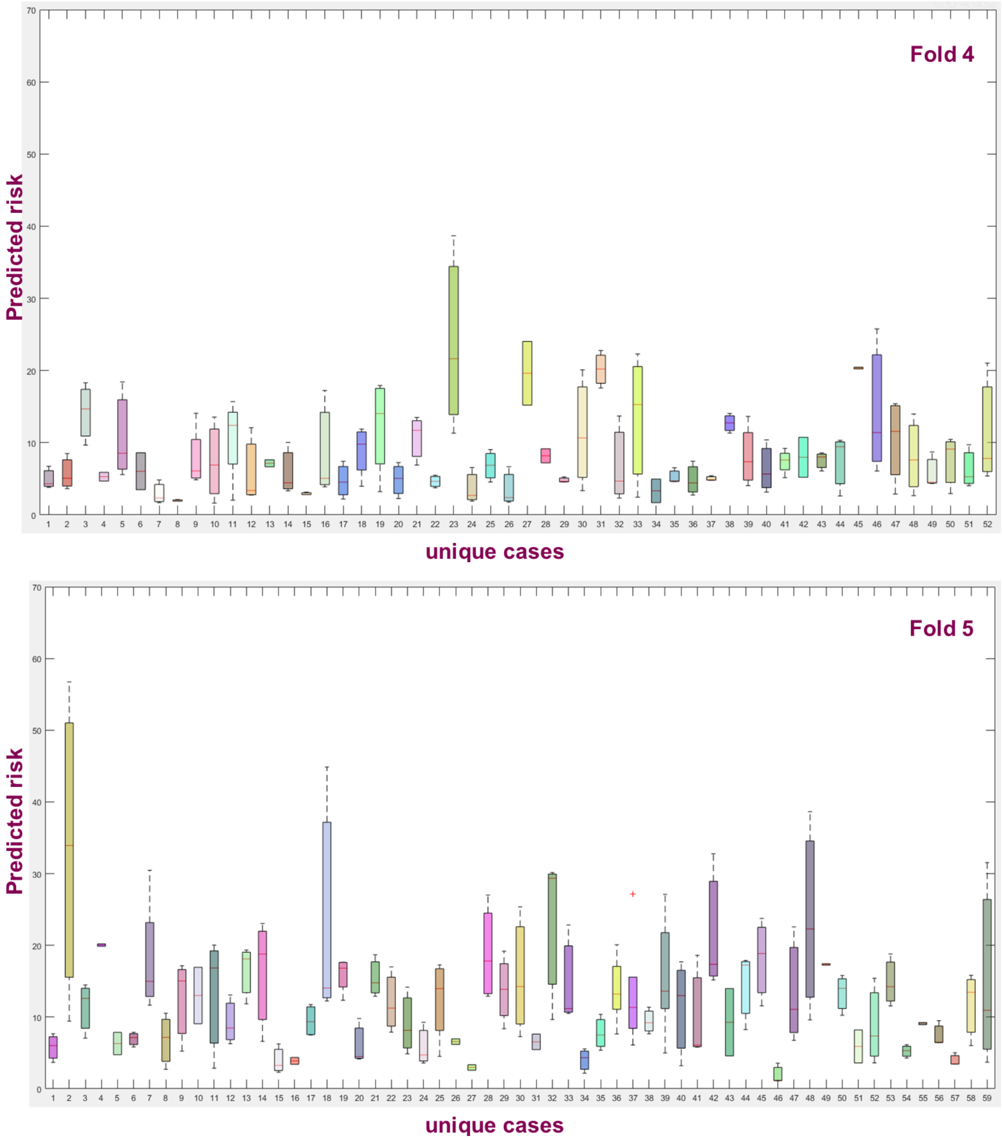

Supplementary Figure 2. The WSI level risk prediction analysis for remaining two folds of the lung data set.
